# P02.112. Mind-body medicine therapies for the depression spectrum: a systematic review

**DOI:** 10.1186/1472-6882-12-S1-P168

**Published:** 2012-06-12

**Authors:** S D'Silva, C Poscablo, R Habousha, B Kligler

**Affiliations:** 1 George Washington University, Washington D.C., USA; 2 Albert Einstein College of Medicine, Bronx, USA; 3 Center for Health and Healing, Beth Israel Medical Center, New York City, USA

## Purpose

This paper aims to systematically compare and contrast the evidence for the use of mind-body therapies to address varying degrees of depressive symptoms in populations with and without other chronic co-morbidities. Systematic literature searches of PubMed (Medline), Embase, CINAHL, and the 7 databases encompassed by Current Contents, Web of Science and Web of Knowledge were conducted from 1966 onward.

## Methods

Studies had to be designed as prospective control-comparison, using a mind-body medicine modality at least 2 weeks long, in an adult population that speaks English, with a sample size > 30, and with depression as a primary or secondary outcome measured on an established scale. Methodological quality was evaluated using the modified Scale for Assessing Scientific Quality of Investigations (SASQI) for Complementary and Alternative Medicine (CAM).

## Results

Of the 2964 papers identified by database searches, 90 met our inclusion and exclusion criteria. Sixty percent of these papers received a SASQI score >9 and were deemed of sufficient quality to be included in the review. Seventy-two percent of these selected quality papers demonstrated positive effects on the improvement of depressive symptoms. Self-regulation (biofeedback, guided imagery and hypnosis) and interventions with mixed modalities had a higher proportion of positive results than movement (yoga, taichi and qigong) and mind-based (meditation and mindfulness) categories, although the latter two categories have been better studied.

## Conclusion

Along with established psychiatric treatments of therapy and medications for depression, the use of evidence-based mind-body therapies can provide further relief of symptoms in a patient-centered manner. The likely long-term increased cost-effectiveness of integrating these therapies deserves further investigation.

